# Supplementary material for: Content-rich biological network constructed by mining PubMed abstracts
Source: BMC Bioinformatics. 2004 Oct 8;5:147. doi: 10.1186/1471-2105-5-147 (PMC528731; doi:10.1186/1471-2105-5-147)
Supplement: Additional File 5 — The original Chilibot query results of the term "long-term potentiation (LTP)" and 22 other terms, limiting the latest references analyzed to the years 1990, 1995, 2000, and 2004. [file 1471-2105-5-147-S5.bz2 › chilibotAdditionalFile5/ltp1995/html/NMDA_SYNAPTOPHYSIN.html]

 


 **NMDA** and **SYNAPTOPHYSIN** 
  
Found 3 abstracts in PubMed,  **3 abstracts were retrieved and analyzed**.  


---

 Search Google  |
 PDF files only 
|  EDU domain only 

---

- Neuroscience, 1995   Non N methyl D aspartate glutamate receptors are responsible for fast excitatory neurotransmission in the mammalian CNS.
  These receptors are rapidly activated and desensitized in the presence of glutamate, and are often further subdivided into alpha amino 3 hydroxy 5 methyl 4 isoxazole proprionic acid and kainate receptors based on their selective agonists.
  Non **NMDA** glutamate receptors are composed of multiple subunits which recently have been cloned, and studies on the recombinant glutamate receptors have helped clarify the distinctions between AMPA and kainate preferring glutamate receptors.
  Although the subunits which make up both AMPA and kainate receptors have a widespread distribution, most currents recorded in vivo are characteristic of recombinant AMPA receptors.
  To help clarify the functional role of high affinity kainate receptors, we have characterized the expression of a high affinity kainate receptor subunit, KA2, in cultured hippocampal neurons.
  Using immunocytochemistry, we found that KA2 was expressed in hippocampal neurons at all times during the development of the cells in culture, and the subunit was enriched in dendritic spines after about 14 days.
  The subcellular distribution of KA2 paralleled that of the AMPA receptor subunit GluR1, with the AMPA and kainate subunits being colocalized at all times in culture.
  The enriched KA2 immunoreactivity co localized with the synaptic vesicle protein **synaptophysin** at the resolution of light microscopy, indicating synaptic localization of KA2.
  Although the kainate subunit KA2 co localized with the AMPA subunit GluR1, co immunoprecipitation experiments demonstrated a direct interaction between the AMPA receptor subunits GluR1 and GluR2 3, but not between GluR1 and the kainate subunits GluR6 7 or KA2.
  We therefore, conclude that both AMPA and kainate receptor subunits are enriched in the same dendritic spines, yet do not combine to form receptor complexes.

  - Hippocampus, 1995   Cultured hippocampal slices retain many in vivo features with regard to circuitry, synaptic plasticity, and pathological responsiveness, while remaining accessible to a variety of experimental manipulations.
    The present study used ligand binding, immunostaining, and in situ hybridization assays to determine the stability of AMPA and **NMDA** type glutamate receptors and other synaptic proteins in slice cultures obtained from 11 day postnatal rats and maintained in culture for at least 4 weeks.
    Binding of the glutamate receptor ligands 3H AMPA and 3H MK 801 exhibited a small and transient decrease immediately after slice preparation, but the binding levels recovered by culture day 5 10 and remained stable for at least 30 days in culture.
    Autoradiographic analyses with both ligands revealed labeling of dendritic fields similar to adult tissue.
    In addition, slices at CD 10 20 expressed a low to high affinity 3H AMPA binding ratio that was comparable with that in the adult hippocampus .
    AMPA receptor subunits GluR1 and GluR2 3 and an **NMDA** receptor subunit exhibited similar postcutting decreases as that exhibited by the ligand binding levels, followed by stable recovery.
    The GluR4 AMPA receptor subunit was not evident during the first 10 CDs but slowly reached detectable levels thereafter in some slices.
    Immunocytochemistry and in situ hybridization techniques revealed adult like labeling of subunit proteins in dendritic processes and their mRNAs in neuronal cell body layers.
    Long term maintenance was evident for other synapse related proteins, including **synaptophysin**, neural cell adhesion molecule isoforms , and an AMPA receptor related antigen , as well as for certain structural and cytoskeletal components .
    In summary, following an initial and brief depression, many synaptic components were expressed at steady state levels in long term hippocampal slices, thus allowing the use of such a culture system for investigations into mechanisms of brain synapses.

    - Pharmacology, 1992   **Activated astrocytes, but not pyramidal cells, increase glucose utilization in rat hippocampal CA1 subfield after ischemia.** .
      The local cerebral glucose utilization CMRglc in the damaged rat hippocampal CA1 subfield increases 7 days after 10 min of cerebral ischemia.
      We have used the N methyl D aspartate antagonist **NMDA** antagonist ketamine in rats 7 days after sham operation or cerebral ischemia to determine whether the elevated postischemic CMRglc of the CA1 subfield is due to long lasting hyperexcitation of surviving or injured neurons, or, alternatively, to the metabolism of other cell types.
      The autoradiographic data were interpreted with the aid of histochemical analysis of the postischemic hippocampal cell changes.
      Anesthetic doses of ketamine significantly reduced the CMRglc in the CA1 strata oriens, pyramidale and radiatum of sham operated rats, while the postischemic increases in CMRglc in these hippocampal CA1 strata were not affected by ketamine.
      In addition, there were ketamine induced increases in the CMRglc of the CA1 stratum lacunosum moleculare of both sham operated and postischemic rats.
      The immunoreactivity of the microtubule associated protein 2 MAP2 , a postsynaptic protein marker, was decreased markedly in the CA1 subfield in postischemic rats, while the presynaptic protein marker, **synaptophysin**, remained the same in sham operated and postischemic rats.
      The glial fibrillary acidic protein GFAP immunoreactivity of astrocytes raised markedly in the ischemically damaged CA1 subfield.
      Although it could be demonstrated that presynaptic terminals remain intact in the postischemic damaged CA1 subfield, the lacking ketamine effect on CA1 pyramidal neurons indicated that the increase in CMRglc in this brain area is not due to postsynaptic neural hyperexcitation, but probably has to be attributed to astrocytes activated by neuronal damage.
